# Physical Activity Enjoyment and Orthorexic Eating Behaviours in Turkish Adults: A Cross-Sectional Study

**DOI:** 10.3390/healthcare14050677

**Published:** 2026-03-07

**Authors:** Bekir Erhan Orhan, Hussain Yasin, Aydın Karaçam, Umut Canlı, Mehdi Ben Brahim

**Affiliations:** 1 Faculty of Sport Sciences, Istanbul Aydın University, Istanbul 34295, Türkiye; 2 Health and Physical Education (HPE) Department, Sport Sciences and Diagnostics (GSD) Research Group Department, Prince Sultan University, Riyadh 11586, Saudi Arabia; hyasin@psu.edu.sa; 3 Faculty of Sports Sciences, Bandırma Onyedi Eylül University, Balıkesir 10200, Türkiye; akaracam@bandirma.edu.tr; 4 Faculty of Sports Sciences, Tekirdağ Namık Kemal University, Tekirdağ 59030, Türkiye; ucanli@nku.edu.tr

**Keywords:** eating behaviours, orthorexia nervosa, physical activity, exercise, diet

## Abstract

**Highlights:**

**What are the main findings?**

- Enjoyment of physical activity was not meaningfully associated with orthorexic tendencies in adults.
- Orthorexic symptoms were modestly related to higher BMI and self-identifying one’s diet as healthy and balanced.

**What are the implications of the main findings?**

- Pleasure derived from physical activity does not appear to be a key factor in orthorexic eating in non-clinical populations.
- Weight status and diet-related self-perceptions may be more relevant targets for understanding and assessing orthorexic tendencies.

**Abstract:**

**Background**: Orthorexic eating reflects a rigid preoccupation with healthy eating that often co-occurs with health-oriented lifestyles, yet the affective experience of physical activity has received little attention. This study examined whether enjoyment of physical activity is associated with orthorexic tendencies in adults and whether it explains variance beyond age, body mass index (BMI), physical activity status, and self-rated diet. **Methods:** Adults (N = 434; M_age = 27.55) recruited online in Türkiye completed a survey including the Physical Activity Enjoyment Scale (PACES), the Orthorexia Nervosa Inventory (ONI), and sociodemographic, BMI, physical activity, and diet items. Pearson correlations and one-way ANOVAs assessed bivariate associations, and hierarchical regressions tested whether PACES added incremental variance to ONI total and domain scores beyond covariates. **Results:** PACES scores showed a near-zero correlation with ONI total (r ≈ 0.02) and did not add variance in regression models (ΔR^2^ ≈ 0.00). Higher BMI and identifying one’s diet as “healthy and balanced” were linked to modestly higher ONI total and Impairments/Emotions scores, while differences in physical activity status were small and mainly limited to the Behavioural domain. **Conclusions:** In this non-clinical sample of Turkish adults, enjoyment of physical activity was not meaningfully associated with orthorexic tendencies. These findings suggest that enjoyment-focused physical activity promotion can be encouraged without increasing orthorexic symptoms, while replication in clinical/high-risk groups (e.g., elite/professional athletes and clinical eating disorder patients) and longitudinal designs is warranted.

## 1. Introduction

Orthorexic eating, characterised by an obsessive preoccupation with consuming foods perceived as “pure,” “clean,” or “healthy,” has attracted growing attention as a potential public health concern within contemporary wellness cultures that strongly valorise dietary control and physical activity [1,2,3]. Although striving for a healthy lifestyle is generally associated with favourable physical and psychological outcomes, the pursuit of dietary “perfection” can become rigid and impairing, leading to nutritional deficiencies, social isolation, and marked distress when self-imposed rules are violated [2,4,5]. In response to conceptual and diagnostic ambiguity, recent work has emphasised orthorexia nervosa as a syndrome involving persistent preoccupation with healthy eating, compulsive behaviours, and clinically relevant impairment, while noting that it is not yet recognised as a formal eating disorder diagnosis [1,2,5,6].

Epidemiological and clinical studies indicate that orthorexic tendencies are present in both clinical and non-clinical populations, but the reported prevalence varies widely depending on sampling strategies and measurement approaches, particularly earlier tools that have been criticised for limited specificity [5,6,7,8]. Across university students, health professionals, and physically active groups, orthorexic symptoms have been linked to dieting, perfectionism, body image concerns, and strong internalisation of health ideals, although effect sizes and patterns of association differ across contexts [9,10]. Recent reviews have therefore highlighted the importance of robust, multidimensional assessment frameworks that distinguish adaptive healthy eating from pathological forms of dietary restraint [4,6,8,11,12]. In this context, instruments such as the Orthorexia Nervosa Inventory (ONI) offer a psychometrically supported multidimensional structure, capturing behavioural features, functional impairment, and emotional distress through Behavioural, Impairments, and Emotions domains [5,13,14,15].

Physical activity occupies a similarly ambivalent position within contemporary health promotion. It is widely endorsed as protective for physical and mental health, yet also features in compulsive exercise and exercise addiction patterns that co-occur with disordered eating [11,12,16]. Cross-sectional work in fitness settings and student samples has reported positive associations between orthorexic symptomatology, exercise volume, and indices of exercise addiction, suggesting that some individuals may combine rigid healthy eating with highly driven or compulsive training behaviours [6,8,9,17,18,19,20,21]. At the same time, many physically active individuals with strong health goals do not show psychopathology, and increasing attention has been directed to the qualitative, affective experience of exercise, such as enjoyment versus obligation, as a potential factor differentiating adaptive high involvement from maladaptive patterns [11,12,16,20]. Within this literature, constructs such as exercise volume, obligatory exercise, and exercise addiction primarily index how much and how compulsively individuals exercise, whereas they do not directly capture the affective quality of exercise participation.

Enjoyment of physical activity represents a central affective construct within motivational frameworks such as self-determination theory and habit-formation models, and is consistently associated with higher intention to be active, better adherence, and more sustainable participation across age groups [11,19]. In contrast to indices of exercise volume or exercise addiction, which quantify how often and how compulsively individuals exercise, enjoyment reflects the positive emotional experience during participation. Higher enjoyment is generally regarded as a marker of adaptive engagement that supports long-term physical activity habits. However, it remains unclear whether the pleasure derived from exercise is inversely related to orthorexic tendencies, by fostering flexible, self-determined health behaviours, or whether, in some individuals, high enjoyment co-occurs with more rigid, perfectionistic investment in “healthy” lifestyles [6,8,17,19].

From a self-determination perspective, enjoyment is typically aligned with autonomous motivation (e.g., interest and personal value), which tends to support behavioural flexibility and reduce reliance on rigid self-control; therefore, higher enjoyment could plausibly be protective against orthorexic rules driven by controlled motives (e.g., guilt, external evaluation, or moralised health norms) [11,12,19]. Conversely, within wellness-oriented subcultures, enjoyment may coexist with strong internalisation of health ideals, perfectionistic standards, or obsessive passion, such that positive affect during exercise accompanies a rigid “clean-living” identity [6,8,16,17]. Habit formation models likewise imply that positive affect strengthens repetition and automaticity of health behaviours; whether this repetition remains adaptive may depend on cognitive flexibility and the extent of functional impairment [11,19].

Despite advances in multidimensional assessment, relatively little is known about how orthorexic symptomatology defined in this way relates to positive exercise-related constructs such as enjoyment, particularly in non-clinical adult populations outside predominantly Western contexts [6,8,20]. Taken together, existing evidence suggests that orthorexic eating is embedded within broader constellations of health-oriented behaviours and motives, but the specific role of physical activity enjoyment within this constellation remains insufficiently understood [18,19,20,22]. Accordingly, the present study addressed two research questions: (1) To what extent is enjoyment of physical activity associated with orthorexic eating behaviours in adults? and (2) Does enjoyment of physical activity explain additional variance in orthorexic tendencies beyond key sociodemographic, anthropometric, and lifestyle characteristics such as age, body mass index, physical activity status, and dietary patterns? By focusing on enjoyment rather than exercise volume or addiction indices, the study evaluates whether the affective quality of exercise adds explanatory value beyond previously studied indicators of quantitative load and compulsive involvement. Given the mixed theoretical expectations regarding whether enjoyment is protective or co-occurs with rigid health investment, no strong directional hypothesis was specified, and the analyses were framed as an initial test of association.

## 2. Materials and Methods

This research employed a cross-sectional survey design in accordance with the STROBE guidelines for observational studies [23] and the CHERRIES checklist for online data collection [24]. The study was conducted through an online questionnaire distributed across Türkiye between July and September 2025. A single, locked form version was used throughout data collection, and responses were anonymous in the analytic export (no names, national IDs, emails, phone numbers, or IPs). The study aimed to examine whether physical activity enjoyment predicts orthorexic eating tendencies, alongside key covariates.

### 2.1. Study Group

Eligible participants were adults aged ≥ 18 years, residing in Türkiye, with sufficient Turkish literacy to complete the survey and willingness to provide electronic informed consent. A total of 520 submissions were received; applying a priori exclusions (age < 18; implausible anthropometrics, BMI < 10 or >60 kg/m^2^; missing beyond pre-specified thresholds for PACES or ONI) yielded an analytic sample of N = 434. Optional non-response was permitted for non-core items, and participants could discontinue at any time without penalty. Participants were recruited via convenience and snowball sampling through university mailing lists, social media, and personal networks. Invitations outlined the study and inclusion criteria, emphasised voluntary, non-incentivised participation, and requested single survey completion to help reduce recruitment bias.

### 2.2. Ethics

The protocol received approval from the Istanbul Aydın University Social and Human Sciences Ethics Committee (Meeting No. 2025/7, 25 June 2025) and complied with the Declaration of Helsinki. Before accessing the survey items, all participants viewed an online information sheet outlining the study purpose, voluntary participation, minimal risks, confidentiality, data use, and withdrawal rights, and provided informed consent by selecting “I agree.” No direct identifiers were collected. De-identified data were stored on password-protected institutional accounts and encrypted drives accessible only to the research team and will be retained for at least five years after publication.

### 2.3. Sociodemographic, Health, and Lifestyle Variables

Participants reported age (years), sex (male/female), marital status (single/married), education (less than high school; high school; university/associate; post-graduate), employment (employed vs. not), and place of residence (province/city). Height (cm) and weight (kg) were used to compute BMI as weight (kg)/height (m)^2^, and categorised using adult WHO thresholds: underweight (<18.5), normal (18.5–24.9), overweight (25.0–29.9), and obese (≥30.0). Weekly physical activity (PA) frequency was assessed as the number of days with moderate-to-vigorous PA in a typical week (response options: never; 1–2; 3–4; 5–6; every day) and grouped a priori as inactive (never), insufficiently active (1–2 days/week), and regularly active (≥3 days/week), consistent with adult PA guidance [25,26]. Diet pattern was assessed with a single global self-report item asking participants to rate their usual eating pattern (‘unbalanced/unhealthy’, ‘partly healthy’, ‘healthy and balanced’). The wording was drafted by the research team to provide a brief, face-valid indicator of perceived diet quality in the context of an online survey and to reflect labels commonly used in public health nutrition messages.

### 2.4. Physical Activity Enjoyment-PACES

The Physical Activity Enjoyment Scale (short form; PACES) assesses the affective component of exercise participation with 8 items rated on a 7-point Likert scale (1 = strongly disagree to 7 = strongly agree). The validated Turkish short form (FAKÖ) was used without modification; all items are positively keyed in the Turkish version and were presented on a single page to minimise order effects. Respondents were instructed to indicate how much they generally enjoy engaging in physical activity/exercise. The total score is the mean of the eight items (range 1–7), with higher values indicating greater enjoyment; no clinical cut-points are defined, so scores are treated as continuous. As pre-specified for missing data, responses with ≤1 missing item were imputed using the respondent’s person-mean; otherwise, the scale score was set to missing for analysis. Internal consistency was evaluated with Cronbach’s α. In our sample, α = 0.96, indicating excellent reliability. The Turkish validation supported a unidimensional structure with excellent model fit (χ^2^/df ≈ 2.37, GFI ≈ 0.98, CFI ≈ 0.99, TLI ≈ 0.99, RMSEA ≈ 0.04–0.05, SRMR ≈ 0.01), very high internal consistency (α ≈ 0.96; CR ≈ 0.95; AVE ≈ 0.74), and strong convergent validity with physical activity behaviour [27]. Cross-cultural adaptation of the Turkish form followed standard procedures (forward/back translation, expert review, pilot cognitive checks), ensuring conceptual, semantic, and operational equivalence to the source instrument [28].

### 2.5. Orthorexic Eating Behaviours-ONI

The Orthorexia Nervosa Inventory (ONI) was administered in its validated Turkish adult form without modification and comprises 24 items rated on a 4-point Likert scale (1 = never, 2 = sometimes, 3 = often, 4 = very often), with higher scores reflecting greater orthorexic symptomatology; the primary outcome was the ONI total score, computed as the sum of all items (range 24–96) and treated as a continuous index of orthorexic eating tendencies. In line with the Turkish validation, three domain scores were also calculated as secondary outcomes, Behavioural (rigid rules, checking/monitoring, avoidance of foods deemed “unhealthy”), Impairments (functional interference with social/occupational life, time burden, attentional and social constraints), and Emotions (anxiety, guilt, emotional rigidity around eating), with higher subscale scores indicating stronger expression of the respective domain. The instrument was self-administered online; rare instances of multiple selections on a single item were resolved conservatively by retaining the rightmost (final) option, and missing data were handled a priori by person-mean imputation when ≤4 of 24 items (≤16.7%) were missing; otherwise, ONI scores were set to missing for analyses requiring that outcome. Internal consistency in the present sample was excellent for the ONI total (α = 0.95) and good to excellent for the subscales (Behavioural α = 0.89; Impairments α = 0.80; Emotions α = 0.85), aligning with the Turkish adult validation, which reported α_total ≈ 0.91 and acceptable three-factor CFA fit (RMSEA ≈ 0.08; CFI ≈ 0.94; NFI ≈ 0.93; SRMR ≈ 0.07; IFI ≈ 0.94), as well as convergent validity with disordered eating risk (EAT-26; r ≈ 0.42) and a small positive association with BMI (r ≈ 0.16) [29,30].

### 2.6. Data Handling, Scoring, and Reliability

Records were screened a priori and excluded if the respondent was <18 years or reported implausible BMI (<10 or >60 kg/m^2^). Rare choice artefacts on ONI items (e.g., multiple options captured in an ONI cell) were resolved by retaining the rightmost token as the final response. Missing data were handled at the scale level using person-mean imputation under a MAR assumption when missingness was small and pre-specified: for PACES, if ≤1/8 items (≤12.5%) were missing; for ONI, if ≤4/24 items (≤16.7%) were missing; otherwise, the corresponding scale score was set to missing for analyses requiring that outcome [31,32]. Two rare free-text diet entries were conservatively mapped to Partly healthy to avoid misclassification while preserving complete-case N. Frequencies and percentages of cases with item-level imputation on PACES and ONI, and of records excluded due to missingness exceeding these thresholds, were recorded during data screening. Scoring followed published guidance: PACES items (1–7) were averaged (higher = greater enjoyment), and ONI items (1–4) were summed to an ONI total (24–96; higher = stronger orthorexic tendencies) with three subscales (Behavioural, Impairments, Emotions) analysed as continuous indices. Reliability was estimated via Cronbach’s α (PACES α = 0.96; ONI total α = 0.95; Behavioural α = 0.89; Impairments α = 0.80; Emotions α = 0.85); given α’s tau-equivalence assumptions, we also inspected item–total correlations and McDonald’s ω (ω_total ≈ 0.96 for PACES; 0.95 for ONI), which led to the same qualitative conclusions [33,34,35]. Model assumptions (residual normality, homoscedasticity, multicollinearity) were checked; VIF < 2.5 indicated no problematic collinearity.

### 2.7. Statistical Analysis

Analyses were conducted in IBM SPSS Statistics 28.0 [36]. Descriptive statistics (means, standard deviations, frequencies, and percentages) summarised sample characteristics. Pearson correlations quantified bivariate associations among continuous study variables. Group differences in ONI scores across physical activity and diet categories were examined using one-way ANOVAs; when the homogeneity-of-variance assumption was violated (Levene’s test), Welch-corrected F tests were used, and Bonferroni-adjusted post-hoc comparisons based on Welch’s t tests were applied to control family-wise error. Conventional ANOVA F values are presented in Table 1, with corresponding Welch-corrected omnibus tests and pairwise contrasts reported in Supplementary Table S2A,B for outcomes with heterogeneous variances. Partial ηp^2^ was reported as the effect-size index for ANOVAs [37]. All tests were two-tailed with α = 0.05.

The primary inferential models were hierarchical linear regressions predicting ONI total and subscale scores. In Block 1, age, BMI, physical activity status, and self-rated diet pattern were entered as covariates. These variables were selected a priori as proximal indicators of body composition and global health investment that have been most consistently linked to orthorexic and disordered eating tendencies and to health-related behaviours. In Block 2, PACES scores were added to evaluate whether enjoyment of physical activity explained additional variance in orthorexic symptoms beyond these covariates (ΔR^2^).

Regression assumptions were evaluated using normality and homoscedasticity plots of residuals, variance inflation factors (VIF < 2.5) and tolerance values for multicollinearity, and standardised residuals and Cook’s distance for influence. Where heteroskedasticity was suggested, models were re-estimated with heteroskedasticity-consistent standard errors, and the substantive conclusions were unchanged [38]. Details of the a posteriori sensitivity analysis, including G*Power 3.1.9.2 (Franz Faul, Universität Kiel, Germany) specifications for the ANOVAs and the incremental ΔR^2^ detectable in the hierarchical regression models, are summarised in the separate Sensitivity analysis subsection and in Table 1 [39,40].

#### Sensitivity Analysis

An a posteriori sensitivity analysis was conducted in G*Power 3.1 to estimate the minimal detectable effect sizes given the realised sample size and α = 0.05. This analysis is reported for transparency and should not be interpreted as observed/post-hoc power providing evidence beyond the *p*-values and confidence intervals. For the one-way ANOVAs with four BMI groups (N = 434), the design was sensitive to small effects of approximately f = 0.10 or larger [39,40]. For the hierarchical regression models (incremental test of PACES in Block 2 with df_1_ = 1, df_2_ ≈ 427), the available sample was sensitive to very small increases in explained variance (ΔR^2^ ≈ 0.015–0.020). Detailed G*Power specifications and detectable effect sizes are summarised in Table 1.

## 3. Results

### 3.1. Sample Characteristics

A total of 434 adults met all inclusion criteria and provided sufficient data for the main analyses. Item-level missingness was handled using the prespecified scale-scoring rules (see Methods). The sample was young-to-mid-adult (age M= 27.55, SD = 10.57), with 55.8% male (*n* = 242) and 44.2% female (*n* = 192). Mean BMI was 24.48 kg/m^2^ (SD = 3.91); 6.0% underweight (*n* = 26), 53.0% normal weight (*n* = 230), 32.5% overweight (*n* = 141), 8.5% obese (*n* = 37). Physical activity (PA) status: inactive 11.8% (*n* = 51), insufficiently active (1–2 days/week) 26.7% (*n* = 116), regularly active (≥3 days/week) 61.5% (*n* = 267). Diet pattern: unbalanced 17.7% (*n* = 77), partly healthy 65.9% (*n* = 286), healthy and balanced 16.4% (*n* = 71). Full sociodemographic and health distributions are presented in Table 2.

The sample included 55.8% male and 44.2% female, was skewed toward normal and overweight BMI categories, and predominantly reported engaging in regular weekly physical activity.

### 3.2. Descriptive Statistics and Reliability of Study Measures

PACES demonstrated excellent internal consistency (α = 0.96; M = 6.00, SD = 1.23). The ONI total also showed excellent reliability (α = 0.95; M = 49.54, SD = 14.05). ONI subscales were Behavioural (M = 23.80, SD = 6.71; α = 0.89), Impairments (M = 9.33, SD = 3.39; α = 0.80), and Emotions (M = 16.40, SD = 5.15; α = 0.85).

*Bivariate Associations among Study Variables*

BMI increased with age (r ≈ 0.41) and showed a small positive association with ONI total (r ≈ 0.11), whereas PACES was essentially uncorrelated with ONI (r ≈ 0.02; Table 3). Across analyses, effect sizes were small (e.g., r ≈ 0.11; ηp^2^ ≈ 0.02–.04; ΔR^2^ ≈ 0.00), indicating limited practical impact at the population level.

### 3.3. Differences in PACES and ONI Scores by BMI Category

Omnibus ANOVA results comparing PACES and ONI outcomes across BMI categories (underweight, normal weight, overweight, and obese) are presented in Table 4. Significant omnibus effects were observed for ONI total and all subscales, whereas PACES did not differ significantly across BMI groups.

### 3.4. Hierarchical Regression Analyses Predicting Orthorexic Symptoms

Across hierarchical regression models, PACES did not add independent explanatory value beyond age, BMI, physical activity status and self-rated diet in predicting ONI total or domain scores (ΔR^2^ ≈ 0.00). Higher BMI and endorsement of a ‘healthy and balanced’ diet were associated with higher scores, whereas the age effect was small and negative (Table 5). Descriptive ONI total and domain scores by diet pattern are presented in Supplementary Table S1 to illustrate the magnitude of these differences.

Supplementary models including sex (and education) as additional covariates yielded a very similar pattern of results, with PACES remaining a non-significant predictor and BMI and self-rated diet pattern retaining their status as the most consistent correlates of ONI scores.

### 3.5. Incremental Contribution of Physical Activity Enjoyment

To evaluate whether physical activity enjoyment explained additional variance in orthorexic tendencies beyond key covariates, hierarchical regressions were conducted in which Model 2 included age, BMI, physical activity status, and self-rated diet, and Model 3 added PACES. The inclusion of PACES yielded negligible incremental explanatory value across ONI subscales (ΔR^2^ ≤ 0.006) and did not independently predict any outcome. Specifically, PACES did not predict Behavioural (R^2^ = 0.131 in Model 2 vs. 0.131 in Model 3; ΔR^2^ = 0.0005; *p* = 0.619), Impairments (R^2^ = 0.054 vs. 0.059; ΔR^2^ = 0.0055; *p* = 0.115), or Emotions (R^2^ = 0.053 vs. 0.053; ΔR^2^ = 0.0003; *p* = 0.726). Overall, adjusted models indicated that the more consistent correlates of ONI dimensions were BMI (particularly for Behavioural and Emotions) and diet self-identification, whereas enjoyment of physical activity contributed no meaningful incremental variance. Full hierarchical model increments are provided in Supplementary Table S3.

### 3.6. Sensitivity Analyses and Model Diagnostics

Complete-case analyses (no imputation) and regressions estimated with HC3 heteroskedasticity-consistent standard errors produced the same pattern of significance and effect directions. Diagnostic plots indicated acceptable residual normality and homoscedasticity, and VIF values were <2.5 throughout. Full estimates for the complete-case models are available from the authors upon reasonable request.

Taken together, the findings indicate that, in this cohort, orthorexic tendencies align more closely with anthropometric status and self-rated diet pattern than with enjoyment of physical activity per se. BMI and diet-related self-perceptions emerged as the most consistent correlates of ONI total and domain scores.

## 4. Discussion

This cross-sectional study examined whether enjoyment of physical activity is associated with orthorexic eating tendencies in adults and whether it explains additional variance in orthorexic symptoms beyond age, body mass index, weekly physical activity status, and self-rated diet. In line with previous work linking orthorexia to broader health- and weight-related characteristics, orthorexic tendencies were more closely aligned with BMI and self-identification with a “healthy and balanced” diet than with affective responses to exercise. Physical activity enjoyment, as measured by PACES, showed correlations with ONI total and domain scores near zero and did not add additional explained variance in hierarchical regression models, indicating no meaningful relationship with orthorexic tendencies in this sample. Taken together, the findings suggest that orthorexic eating in this adult Turkish sample is embedded more strongly in patterns of weight status and diet-related self-perception than in the subjective pleasure derived from physical activity. Because the present data derive from a non-clinical adult sample recruited online in Türkiye, these patterns may not generalise to clinical eating disorder populations, elite or professional athletes, or individuals with very high exercise volumes or pronounced exercise addiction.

The absence of an independent association between physical activity enjoyment and ONI scores contributes to ongoing debates about the role of exercise within orthorexic and other health-oriented eating patterns [11,12,16]. Prior studies have typically focused on exercise volume, obligatory exercise, or exercise addiction, often finding positive associations with orthorexic tendencies in fitness and student [9,17,18,19,20,21,41,42]. In contrast, the present study isolated a specific affective component of physical activity engagement, enjoyment, and found that PACES scores did not provide evidence that higher enjoyment is associated with higher or lower orthorexic tendencies once BMI, physical activity status, and diet are accounted for. From a health promotion perspective, this pattern implies that strategies designed to increase intrinsic pleasure and enjoyment in physical activity are unlikely to trigger or exacerbate orthorexic symptomatology in community-dwelling adults, although they should not be assumed to prevent orthorexia in higher-risk groups.

BMI emerged as a small but consistent correlate of orthorexic tendencies, with overweight and obese participants reporting slightly higher ONI scores than normal-weight and underweight peers. Although effect sizes were modest, this pattern aligns with research linking orthorexic symptoms and weight-related concerns in community and at-risk groups [7,8,22,43]. The present findings are compatible with at least two interpretations. On the one hand, individuals with higher BMI may experience greater body dissatisfaction and health worries, which could foster investment in “clean” or “healthy” eating as a perceived route to weight control or risk reduction. On the other hand, some individuals with pronounced orthorexic tendencies may show weight trajectories that do not mirror their strict dietary rules—for example, because rigid eating alternates with episodes of overeating or because weight is influenced by metabolic, genetic, or medication-related factors. The cross-sectional design does not allow these pathways to be disentangled, but the small effect sizes and modest R^2^ values caution against viewing BMI as a strong or deterministic indicator of orthorexic risk. In particular, weight-related distress and internalised weight stigma may motivate stricter “clean-eating” rules as a compensatory attempt to manage perceived health risk or body concerns, whereas metabolic, medication-related, or compensatory eating processes may also contribute to BMI trajectories that are not directly proportional to dietary rule adherence.

Self-rated diet pattern showed the clearest association with ONI scores. Participants who described their diet as “healthy and balanced” reported higher orthorexic tendencies than those labelling their diet as unbalanced/unhealthy or partly healthy. Because this measure reflects self-perceived diet quality rather than an objective assessment of nutritional intake, higher ONI scores in this group may index identity-based endorsement of healthy eating ideals rather than genuinely superior dietary patterns. Notably, the single-item diet label is subjective and may share conceptual overlap with ONI content (e.g., moralised “healthy eating” identity); this subjectivity could inflate the observed association between endorsing a “healthy and balanced” diet and higher ONI scores.

This is broadly consistent with work emphasising that orthorexia is anchored in an intense identification with healthy eating ideals and strict dietary rules, rather than in obvious dietary neglect [4,5,6]. At the same time, the present study relied on a single global self-rated diet item, which likely captures respondents’ subjective alignment with “healthy eating” norms rather than objective diet quality. Individuals with rigid, perfectionistic standards may be especially inclined to endorse a “healthy and balanced” label even when their pattern involves substantial restriction, moralisation of food, or social impairment. In this sense, the ‘healthy and balanced’ response category may function more as a marker of identity-driven endorsement of healthy eating ideals than of objectively optimal nutritional composition. Thus, while the association between self-labelled “healthy” diet and higher ONI scores reinforces the centrality of health-focused identity in orthorexia, it should be interpreted cautiously and not taken as evidence that nutritionally balanced diets are inherently problematic. More granular assessments of dietary intake, motivations, and rule-bound behaviours will be important to clarify how self-perceived healthy eating maps onto orthorexic pathology.

Domain-specific analyses of the Orthorexia Nervosa Inventory provide additional nuance. Differences by physical activity status were evident only in the Behavioural domain, and even here, the effects were small. This suggests that more frequent physical activity may be linked to a slightly greater enactment of healthy eating behaviours (e.g., planning, monitoring, adherence to rules), without necessarily translating into higher levels of impairment or emotional distress. This pattern aligns with the broader literature in which many individuals with high exercise involvement show structured eating routines that remain functionally adaptive [8,18,20]. The more consistent associations of BMI and diet with Impairments and Emotions emphasise that the adverse impact of orthorexic tendencies, rather than the mere presence of health-oriented practices, is more closely tied to weight status and subjective dietary identity. Such differentiation between behavioural implementation and psychosocial cost is important for distinguishing high but adaptive investment in health from clinically relevant orthorexia nervosa [5,15].

A further contribution of this study lies in the use of a multidimensional, psychometrically robust assessment of orthorexic tendencies in combination with a validated short measure of physical activity enjoyment in a general adult sample. The ONI captures behavioural, impairment-related, and emotional facets of orthorexia with good reliability and evidence for a hierarchical structure [5,14,15,44]. Applying such an instrument in a non-clinical Turkish context adds to growing evidence that orthorexic phenomena can be meaningfully characterised beyond simple cut-offs or single behavioural indicators, and that patterns of association with health-related variables can differ across domains [4,8,13]. In parallel, the PACES provides a concise, affect-focused index of exercise enjoyment that is distinct from exercise volume, compulsive tendencies, or performance motives. The combination of these tools allowed the present analyses to show that, under conditions of adequate statistical power, the association between enjoyment and orthorexia is likely to be trivial in magnitude.

The findings have tentative implications for practice. These implications apply primarily to community-dwelling adults similar to those who participated in this study. From an exercise and health promotion perspective, the results suggest that fostering positive affective experiences during physical activity is unlikely, in itself, to be a major risk marker for orthorexic eating in community adults. Enjoyment-oriented approaches to physical activity—such as providing autonomy-supportive environments, varied and enjoyable activities, and a focus on intrinsic satisfaction—may therefore be pursued without strong concern that they will exacerbate rigid, health-focused eating patterns, although they should not be assumed to prevent such patterns either. It remains uncertain whether the same pattern would hold in clinical samples with established eating disorders, in elite or professional athletes, or in individuals engaging in very high or compulsive volumes of exercise. At the same time, the small but consistent associations with BMI and particularly with self-identification as eating a “healthy and balanced” diet underscore the importance of attending to how individuals make sense of their health behaviours. In people who strongly emphasise dietary purity, present with weight-related distress, or report social and functional interference from their eating rules, clinicians and practitioners may consider a more detailed assessment of orthorexic symptoms, regardless of how much they enjoy physical activity [2,22,45].

For clinicians and dietitians working with clients focused on healthy eating and exercise, these findings highlight the value of exploring not only what and how much individuals eat and move, but also the psychological meaning, flexibility, and social impact of their health routines [4,5,6,20]. Screening for orthorexic tendencies may be particularly relevant when a person presents with a strong identity investment in “clean” or “balanced” eating, experiences guilt or anxiety when deviating from self-imposed rules, or reports strain in social and family functioning due to dietary practices, irrespective of BMI or exercise enjoyment. The current results suggest that a lack of enjoyment in physical activity is not a necessary feature of orthorexia, and that interventions targeting orthorexic symptoms should focus more on cognitive rigidity, emotional distress, and functional impairment around food and health behaviour than on modifying exercise-related affect per se.

The covariate set in the regression models was intentionally restricted to age, BMI, physical activity status, and self-rated diet, as proximal indicators of body composition and global health investment. Other available sociodemographic characteristics (such as sex, education, marital status, and employment status) were not included and may account for additional variance in orthorexic tendencies. Future studies with larger and more diverse samples could incorporate a broader range of sociodemographic indicators to more fully characterise potential confounding and effect modification.

Overall, this study adds to the emerging literature indicating that orthorexic eating is situated within constellations of health-oriented behaviours, weight-related characteristics, and internalised ideals, while also showing that not all positive health-related constructs are strongly implicated. In particular, the findings support a conceptual separation between affective enjoyment of physical activity and the pathological pursuit of dietary purity, and they emphasise that small but consistent associations with BMI and self-labelled healthy eating warrant careful, context-sensitive interpretation rather than simple risk labelling. At the same time, reliance on self-reported behaviours and a convenience, self-selected sample constrains external validity, the characteristics of the study group are likely to over-represent younger, more educated, and health-interested adults. Consequently, the prevalence and patterning of orthorexic tendencies observed here may not fully reflect the broader Turkish adult population, particularly older adults, individuals with lower educational attainment, or those less engaged with health-related issues.

### Strengths and Limitations

This study has several strengths. First, it addresses an under-examined question, whether enjoyment of physical activity is associated with orthorexic tendencies, in a relatively large adult sample using a multidimensional orthorexia measure and a validated enjoyment scale. Second, the analytic strategy combined group comparisons with hierarchical regression and explicit covariate control, and model assumptions were checked, supporting the robustness of the inferences within the limits of observational data.

Several limitations warrant emphasis. First, the diet pattern was assessed with a single global self-report item; this measure likely captured perceived diet identity rather than objective nutritional quality, and the shared emphasis on “healthy eating” may have inflated the diet-ONI association through common-method and construct overlap. Future studies should incorporate validated dietary quality indices (e.g., FFQ/recall-based measures, Mediterranean diet adherence scores) and assess motivations and cognitive rigidity surrounding eating. Second, the convenience, self-selected online sample was skewed toward younger, more educated and regularly active adults (61.5% regularly active), which limits external validity; accordingly, the present estimates may not generalise to sedentary populations, older adults, or clinical/high-risk groups (e.g., elite/professional athletes and clinical eating-disorder patients). Specifically, generalisability may be further constrained in elite/professional athletes, individuals in weight-sensitive sports or occupations, and clinical eating disorder populations. Third, the cross-sectional design precludes causal inference. In addition, key variables were self-reported (including height and weight used to compute BMI), which may have introduced measurement error and social desirability bias. Finally, the a posteriori sensitivity analysis should be interpreted as describing detectable effects in this dataset rather than as prospective power for study planning.

## 5. Conclusions

This cross-sectional study examined whether enjoyment of physical activity is associated with orthorexic eating tendencies in adults and whether it explains additional variance in orthorexic symptoms beyond age, BMI, weekly physical activity status, and self-rated diet. Orthorexic tendencies were more closely aligned with BMI and the self-identification of one’s diet as “healthy and balanced” than with physical activity enjoyment. PACES scores were essentially unrelated to ONI total and domain scores, and adding enjoyment to multivariable models did not meaningfully increase explained variance. These findings suggest that affective enjoyment of physical activity is unlikely to be a major correlate of orthorexic symptoms in non-clinical adults and appears conceptually distinct from more compulsive or obligation-driven patterns of exercise. However, because the sample was recruited online and over-represented younger, more educated and regularly active adults, generalisability to sedentary populations and older adults should be interpreted cautiously.

At the same time, the associations observed with BMI and subjectively “healthy” diet were small in magnitude, and the overall proportion of variance in orthorexic tendencies explained by the models was modest. The cross-sectional, self-report design and reliance on a single global diet item further limit causal inference and the precision of dietary characterisation. Future research should integrate multidimensional measures of orthorexia with more detailed assessments of diet, exercise volume, and compulsive exercise, and employ longitudinal or experimental designs to clarify temporal and causal relationships. For practice, the results tentatively indicate that promoting enjoyable physical activity experiences can remain a central target of health promotion without strong concern that this, in itself, will exacerbate orthorexic tendencies, while clinical attention may be better focused on rigid dietary rules, functional impairment, and distress surrounding “healthy” eating.

## Figures and Tables

**Table 1:** G*Power specifications and sensitivity estimates for ANOVAs and hierarchical regression.

Analysis	Test Family	Statistical Test	Type of Sensitivity Analysis	α	Effect Size Input	Groups/Predictors	Total N	Power at Specified Effect Size
PA (ANOVA)	F tests	ANOVA: Fixed effects, omnibus	Sensitivity: power at specified α, n, f	0.05	f = 0.10 (small)	k = 3	434	≥0.95
BMI (ANOVA)	F tests	ANOVA: Fixed effects, omnibus	Sensitivity	0.05	f = 0.10 (small)	k = 4	434	≥0.90
ANOVA (observed)	F tests	ANOVA: Fixed effects, omnibus	Sensitivity	0.05	f = 0.17–0.20 (from ηp^2^ = 0.029–0.039)	k = 3–4	434	>0.99
Regression (ΔR^2^ = 0.015)	F tests	Linear multiple regression: Fixed model, R^2^ increase	Sensitivity	0.05	f^2^ = 0.015	tested = 1; total = 6	434	≈0.93
Regression (ΔR^2^ = 0.020)	F tests	Linear multiple regression: Fixed model, R^2^ increase	Sensitivity	0.05	f^2^ = 0.020	tested = 1; total = 6	434	≈0.97

Notes. Cohen’s benchmarks: f = 0.10 (small), 0.25 (medium); f^2^ = 0.02 (small), 0.15 (medium). Conversion used f = √[η_p_^2^/(1 − η_p_^2^)]. Degrees of freedom for regression were df_1_ = 1, df_2_ ≈ 427.

**Table 2:** Sociodemographic and health characteristics (N = 434).

Variable	Category	n	%
**Age group**	18–24/25–34/35–44/≥45	266/59/62/47	61.3/13.6/14.3/10.8
**Sex**	Male/Female	242/192	55.8/44.2
**BMI**	Underweight/Normal/Overweight/Obese	26/230/141/37	6.0/53.0/32.5/8.5
**PA status**	Inactive/Insufficient/Regular	51/116/267	11.8/26.7/61.5
**Diet**	Unbalanced/Partly healthy/Healthy and balanced	77/286/71	17.7/65.9/16.4

**Table 3:** Descriptives and correlations among core variables (N = 434).

Measure	M	SD	Age	BMI	PACES	ONI Total
**Age (years)**	27.55	10.57	—	0.409 ***	0.053	−0.043
**BMI (kg/m^2^)**	24.48	3.91		—	0.005	0.111 *
**PACES (1–7)**	6.00	1.23			—	0.017
**ONI total (24–96)**	49.54	14.05				—

Note. Pearson r. *p* < 0.05 *, *p* < 0.001 ***.

**Table 4:** Omnibus ANOVAs by BMI category on PACES and ONI outcomes (N = 434).

Outcome	df (Between, Within)	F	*p*	ηp^2^
**PACES**	(3, 430)	1.01	0.389	0.007
**ONI total**	(3, 430)	4.83	0.003	0.029
**Behavioural**	(3, 430)	4.39	0.005	0.030
**Impairments**	(3, 430)	3.10	0.027	0.021
**Emotions**	(3, 430)	5.89	<0.001	0.039

Notes. BMI: Underweight; Normal; Overweight; Obese. Omnibus F statistics and degrees of freedom shown in Table 4 refer to conventional one-way ANOVAs; Welch-corrected omnibus tests and Bonferroni-adjusted Welch pairwise contrasts for outcomes with heterogeneous variances are reported in Supplementary Table S2A,B. Effect sizes are partial ηp^2^.

**Table 5:** Hierarchical linear regression predicting ONI total (N = 434). Coding: Diet uses Healthy and balanced as reference; PA uses Inactive as reference. Estimation: OLS; two-tailed α = 0.05; HC3 checks confirmed unchanged inferences.

Predictor			B	SE	*p*		95% CI
**Intercept**			43.16	5.55	<0.001		32.26, 54.06
**Age (years)**			−0.17	0.07	0.015		−0.31, −0.03
**BMI (kg/m^2^)**			0.68	0.19	<0.001		0.31, 1.05
**PA: Insufficient vs. Inactive**			1.82	2.42	0.453		−2.93, 6.56
**PA: Regular vs. Inactive**			2.53	2.29	0.271		−1.98, 7.03
**Diet: Partly vs. Healthy**			−5.26	1.80	0.004		−8.81, −1.71
**Diet: Unbalanced vs. Healthy**			−11.11	2.37	<0.001		−15.76, −6.46
**PACES (mean score)**			−0.37	0.57	0.511		−1.49, 0.74
Model fit and change (incremental ΔR^2^)							
**Model (Blocks)**	**Model 1**	**Model 2**				**Model 3**	
**R^2^**	0.0019	0.0797				0.0806	
ΔR^2^ (M2–M1; M3–M2)	—	0.0778				0.0009	
Added predictors	Age	+BMI +PA +Diet				+PACES	
Interpretation	Trivial R^2^	Main variance added (ΔR^2^ ≈ 0.078)				Negligible added variance (ΔR^2^ ≈ 0.001; ns)	
Key message	Age-only model explains ~0%	BMI/PA/Diet explain ~8%				PACES adds ~0%	

Note. Diet uses “healthy and balanced” as the reference category; physical activity (PA) uses “inactive” as the reference category. B = unstandardised regression coefficient; 95% CI = 95% confidence interval for B. Models were estimated using hierarchical linear regression with two-tailed α = 0.05; heteroskedasticity-robust (HC3) checks confirmed unchanged inferences.

## Data Availability

The data presented in this study are available upon request from the corresponding author due to confidentiality concerns.

## Supplementary Materials

The following supporting information can be downloaded at: https://www.mdpi.com/article/10.3390/healthcare14050677/s1. Table S1: ONI subscale descriptives (M, SD, range) and internal consistency (Cronbach’s α) by domain. Table S2A: Bonferroni-adjusted Welch pairwise contrasts for ONI outcomes by physical activity group. Table S2B: Bonferroni-adjusted Welch pairwise contrasts for ONI outcomes by BMI category. Table S3: Incremental variance explained by PACES over demographic and lifestyle covariates for ONI subscale outcomes (Behavioural, Impairments, Emotions).

## Abbreviations

The following abbreviations are used in this manuscript:

BMI	Body Mass Index
ON	Orthorexia Nervosa
ONI	Orthorexia Nervosa Inventory
PACES	Physical Activity Enjoyment Scale
PA	Physical Activity
WHO	World Health Organization
