# Supplementary material for: Physical Activity Enjoyment and Orthorexic Eating Behaviours in Turkish Adults: A Cross-Sectional Study
Source: Healthcare (Basel). 2026 Mar 7;14(5):677. doi: 10.3390/healthcare14050677 (PMC12984885; doi:10.3390/healthcare14050677)
Supplement: Supplementary file 1 [file healthcare-14-00677-s001.zip › healthcare-4151413-supplementary.pdf]

Physical Activity Enjoyment and Orthorexic Eating Behaviors in Turkish Adults: A Cross-Sectional Study

Supplementary Tables

Supplementary Table S1: ONI subscales descriptives (M, SD, range) and internal consistency (α), N = 434.

Subscale	Mean	SD	Range	Cronbach's α
**Behavioral**	23.80	6.71	observed	.89
**Impairments**	9.33	3.39	observed	.80
**Emotions**	16.40	5.15	observed	.85

Note. Higher values indicate stronger expression of each domain. Subscale scores were treated as continuous; no diagnostic cut-points were imposed. Reliability was estimated with coefficient α using listwise deletion at the scale level. Subscale descriptives and α values are summarized in Supplementary Table S1.

All three ONI domains showed good–excellent internal consistency (α = .80–.89). The Behavioral and Emotions domains exhibited larger dispersion than Impairments, consistent with broader behavioral and affective variability in nonclinical adult samples.

Supplementary Table S2A: PA status—pairwise post-hoc comparisons (Bonferroni-adjusted Welch tests).

Outcome	Inactive vs Insufficient	Inactive vs Regular	Insufficient vs Regular
**ONI total**	ns	ns	ns
**Behavioral**	ns	p_adj < .01 (Inactive > Regular)	ns
**Impairments**	ns	ns	ns
**Emotions**	ns	ns	ns

Omnibus: PACES, $F(2,431)=27.73$, $p<.001$, $\eta p^2=.12$; ONI total, $F(2,431)=3.00$, $p=.051$; Behavioral, $F(2,431)=7.21$, $p=.00083$; Impairments, $p=.324$; Emotions, $p=.219$.

Only the Behavioral domain differed by PA status after adjustment for multiplicity, with Inactive > Regular; other pairwise contrasts were non-significant. The corresponding Bonferroni-adjusted pairwise comparisons are shown in Supplementary Table S2A.

Supplementary Table S2B: BMI categories—pairwise post-hoc comparisons (Bonferroni-adjusted Welch tests).

Outcome	Underweight vs Normal	Underweight vs Overweight	Underweight vs Obese	Normal vs Overweight	Normal vs Obese	Overweight vs Obese
**ONI total**	ns	p_adj < .05 (Overweight > Underweight)	ns	p_adj < .05(Overweight > Normal)	ns	ns
**Behavioral**	ns	p_adj < .05 (Overweight > Underweight)	ns	p_adj < .05(Overweight > Normal)	ns	ns
**Impairments**	ns	ns	ns	p_adj < .05(Overweight > Normal)	ns	ns
**Emotions**	ns	p_adj < .01 (Overweight > Underweight)	ns	p_adj < .01(Overweight > Normal)	ns	ns

Omnibus: ONI total, $F(3,430)=4.83$, $p=.003$, $\eta p^2 \approx .029$; Behavioral, $F(3,430)=4.39$, $p=.0047$, $\eta p^2 \approx .030$; Impairments, $F(3,430)=3.10$, $p=.0265$, $\eta p^2 \approx .021$; Emotions, $F(3,430)=5.89$, $p=.00061$, $\eta p^2 \approx .039$.

The Overweight group showed small, consistent elevations versus Normal (and vs Underweight for Behavioral/Emotions). Obese was not reliably different from Normal on these outcomes in this sample after Bonferroni correction.

Pairwise tests used Welch's t with Bonferroni adjustment within each family (PA: 3 contrasts; BMI: 6 contrasts). Only significant contrasts are annotated with direction; all others are ns (non-significant, p_adj ≥ .05).

PA status showed large differences in PACES and a small effect on ONI Behavioral scores only; ONI total and the other subscales did not differ reliably by PA group after adjustment for multiplicity. By contrast, BMI category showed small but consistent elevations in ONI total, Behavioral, and Emotions scores, driven largely by higher scores in the overweight group compared with normal weight (Supplementary Tables S2A–S2B).

Supplementary Table S3: Incremental value of PACES after age, BMI, PA, and diet (N = 434).

Outcome	R^2 (Model 2)	R^2 (Model 3 = +PACES)	ΔR^2	p(PACES)
**Behavioral**	.131	.131	.0005	.619
**Impairments**	.054	.059	.0055	.115
**Emotions**	.053	.053	.0003	.726

Note. R^2 (Model 2) = variance explained by age, BMI, physical activity status and self-rated diet; R^2 (Model 3) = variance explained after adding PACES; ΔR^2 = incremental variance explained by PACES.
